# Research priorities for the study of atrial fibrillation during acute and critical illness: recommendations from the Symposium on Atrial Fibrillation in Acute and Critical Care

**DOI:** 10.1186/s12919-024-00309-x

**Published:** 2024-11-05

**Authors:** Stephanie Sibley, Clare Atzema, Martin Balik, Jonathan Bedford, David Conen, Tessa Garside, Brian Johnston, Salmaan Kanji, Camron Landry, William McIntyre, David M. Maslove, John Muscedere, Marlies Ostermann, Frank Scheuemeyer, Andrew Seeley, Marco Sivilotti, Jennifer Tsang, Michael K. Wang, Ingeborg Welters, Allan Walkey, Brian Cuthbertson

**Affiliations:** 1 Department of Critical Care Medicine, Queen’s University, 76 Stuart Street, Kingston, ON K7L 2V7 Canada; 2 Department of Medicine, University of Toronto, Toronto, Canada; 3 Sunnybrook Research Institute, Toronto, Canada; 4 Department of Anesthesiology and Intensive Care, 1st Faculty of Medicine, Charles University, Prague, Czechia; 5 University of Oxford Nuffield Department of Clinical Neurosciences, Oxford, UK; 6 Population Health Research Institute, McMaster University, Hamilton, Canada; 7 University of Sydney, Royal North Shore Hospital, Sydney, Australia; 8 The George Institute for Global Health, Sydney, Australia; 9 Institute of Life Course and Medical Sciences, Faculty of Health, and Life Sciences, University of Liverpool, Liverpool, UK; 10 Liverpool Centre for Cardiovascular Science, University of Liverpool, Liverpool John Moores University and Liverpool Heart & Chest Hospital, Liverpool, UK; 11 The Ottawa Hospital Research Institute, Ottawa, Canada; 12 Division of Critical Care Medicine, Temerty Faculty of Medicine, University of Toronto, Toronto, Canada; 13 King’s College London, Guy’s & St Thomas’ Hospital London, London, UK; 14 Department of Emergency Medicine, University of British Columbia, Vancouver, Canada; 15 Department of Emergency Medicine, Queen’s University, Kingston, Canada; 16 Niagara Health Knowledge Institute, Niagara Health, St. Catharines, Canada; 17 Department of Medicine, McMaster University, Hamilton, Canada; 18 Division of Health Systems Science, Department of Medicine, University of Massachusetts Chan Medical School, Worcester, MA USA; 19 Department of Anesthesiology and Pain Medicine, Temerty Faculty of Medicine, University of Toronto, Toronto, Canada; 20 Management and Evaluation, Institute for Health Policy, University of Toronto, Toronto, Canada

**Keywords:** Atrial fibrillation, Critical Illness, Research

## Abstract

Atrial fibrillation (AF) is a common arrhythmia encountered in acute and critical illness and is associated with poor short and long-term outcomes. Given the consequences of developing AF, research into prevention, prediction and treatment of this arrhythmia in the critically ill are of great potential benefit, however, study of AF in critically ill patients faces unique challenges, leading to a sparse evidence base to guide management in this population. Major obstacles to the study of AF in acute and critical illness include absence of a common definition, challenges in designing studies that capture complex etiology and assess causality, lack of a clear outcome set, difficulites in recruitment in acute environments with respect to timing, consent, and workflow, and failure to embed studies into clinical care platforms and capitalize on emerging technologies. Collaborative effort by researchers, clinicians, and stakeholders should be undertaken to address these challenges, both through interdisciplinary cooperation for the optimization of research efficiency and advocacy to advance the understanding of this common and complex arrhythmia, resulting in improved patient care and outcomes. The Symposium on Atrial Fibrillation in Acute and Critical Care was convened to address some of these challenges and propose potential solutions.

## Introduction

Atrial fibrillation (AF) is the most common arrhythmia encountered during acute and critical illness [1, 2]. It occurs in many acute care settings, [3] in association with a broad spectrum of medical, surgical, and traumatic conditions [4–9]. AF in the context of acute illness can be problematic as it may be difficult to terminate or control, [10] may worsen hemodynamic instability, and is associated with stroke, [11, 12] thromboembolic events [13–15], and death [16]. The risk of adverse outcomes following newly identified AF persists for years after discharge from hospital, with an elevated risk of developing persistent or recurrent AF [17] as well as hospital readmission for AF, ischemic stroke, heart failure, and death years after discharge [18, 19].

Recent surveys report significant variations in physician practice in the management of AF in these patients, as well as deviation in practice from clinical guidelines [20, 21]. These discrepancies suggest a lack of clarity, acceptability and generalizability of existing AF guidelines to AF in the setting of critical illness, and highlight the paucity of high-quality evidence to inform the management of this arrhythmia in the acute setting. Clinicians must extrapolate from populations who differ with regards to etiology [22], pathophysiology, commonly prescribed treatments, and tolerance to those treatments. The scarcity of data results in missed opportunities for useful interventions, and may expose patients to ineffective and potentially harmful treatments.

Research on AF in acute and critical illness presents unique challenges that hinder attempts to advance our understanding in this field. We convened a symposium to identify and overcome the barriers that limit progress in this important field of research. The specific aim of this symposium was to *address the intersectionality of acute and critical illness, atrial fibrillation, and short and long-term outcomes, with specific focus on inter-professional and inter-disciplinary collaboration aimed at optimizing research efficiency and rigour, encouraging collective engagement, and ultimately improving patient outcomes.*

## The Symposium

The Symposium on Atrial Fibrillation in Acute and Critical Care was held on November 27th and 28th, 2023 in a hybrid format, with an in-person meeting in Toronto, Ontario, Canada and a virtual component that enhanced international participation. Attendees were scientists with an active program of research in AF in acute or critical illness. Members of the working group represented critical care, emergency medicine, pharmacy, anesthesiology, internal/perioperative medicine, cardiology, and thoracic surgery.

Participants presented AF research studies with a focus on *issues or controversies* that present unique challenges. Moderated discussions facilitated thoughtful exchange as to how these issues and controversies impact the design, conduct, and outcomes of research in this field, as well as proposals regarding solutions and strategies to guide future research.

## Issues and controversies

### How is AF defined and categorized during acute and critical illness?

Most studies define AF as a chaotic supraventricular arrhythmia with variable ventricular response resulting in irregularly irregular R-R intervals. Patients can be categorized by the chronicity of their AF; some will have chronic AF with a rapid ventricular response in the setting of acute illness; others have known paroxysmal AF with a recurrence during their illness; some will develop “new-onset” AF if the arrhythmia has not been previously diagnosed. Further classification based on potential pathophysiology must be considered, appreciating the differences in etiology between patients with medical versus surgical illnesses, and cardiac vs non-cardiac surgery patients. Some studies have extended this classification to be syndrome or surgery specific [5–8, 23]. Additionally, severity of illness is a consideration, as the risk factors, treatments, and outcomes of a patient with an acute but hemodynamically stable illness on a medical ward will differ from a hemodynamically unstable patient admitted to an intensive care unit (ICU).

There are no universally accepted definitions of “clinically important” nor “hemodynamically stable/unstable” AF in acute and critical illness. Notably, there is no agreed upon duration of arrhythmia that has been found to be clinically important in the setting of critical illness due to a lack of prospective studies examining the association between duration of arrhythmia and short and long-term outcomes. As a result, studies variably recruit patients with durations of AF ranging from seconds to days, possibly excluding important groups with short runs of AF [24]. Confusion also exists regarding inclusion criteria: patients with known paroxysmal AF who are in sinus rhythm at presentation and patients with chronic AF and rapid ventricular response are variably included in studies of AF in acute and critical illness [1, 24]. Patients with atrial flutter are sometimes included in AF trials [24], and It is unclear if atrial flutter should combined with study of AF, or if it is a different enteity and should be considered separately. This heterogeneity/variability influences the reported epidemiology of AF in acute illness, as the denominator has often undergone variable exclusions prior to the calculation of incidence rates.

- *Research impact - *Research is more efficient and effective when the target condition is clearly and operationally defined. While a common definition would reduce heterogeneity in studies, the trade-off is that a single definition is unlikely to meet all research and clinical needs. AF may require a conceptual definition to frame the research, as well as a more flexible and pragmatic operational definition to enable comparison between studies.
- *Recommendation - *A working group to focus on definitional issues (such as clinically-important, duration, new-onset/first-detected, means of diagnosing) in AF should be convened to establish clear definitions for AF incidence and severity occurring during acute and critical illness. These definitions must consider etiological, concurrent, and pronostic factors and be acceptable and versatile enough to have utility in future studies.

### What is the etiology of AF in acute and critical illness?

AF is an arrhythmia with a complex pathophysiology that is multi-factorial [25]. Risk factors such as advanced age, male sex, and a history of cardiovascular disease [26] suggest that atrial remodeling may precede critical illness, and these structural changes predispose the patient to AF. For some patients, inflammation during acute illness may induce rapid myocardial remodeling that predisposes patients to AF [27]. Identified AF risk factors of vasopressor and inotrope use, electrolyte abnormalities, fluid overload, and high illness severity also suggest adrenergic surge, clinical interventions, and inflammation as contributing arrhythmogenic triggers [26]. Post-operative patients have unique and surgically related risk factors that predispose them to arrhythmia [28]. Translational studies of genomic, transcriptomic, proteomic, and metabololmic pathophysiology of AF in critical illness is scarce. Our understanding of all these factors, and how they interact in the setting of acute and critical illness is limited.

- *Research impact* - AF in acute and critical illness likely arises from combinations of chronic and acute structural remodeling and arrhythmogenic triggers. This may result in heterogeneity in treatment effects and associated outcomes complicating research efforts. Some acute risk factors are potentially reversible during the acute and recovery phases of critical illness and may be useful interventional targets for future interventional studies.
- *Recommendations* - Further study is required for the identification of important etiological risk factors for AF and how these relate to outcomes, with an emphasis on the interactions of patient risk factors, risk from various disease states and severity, sex and gender considerations, and modifiable risk from medical interventions. Biologic heterogeneity adds complexity to clinical research and calls for embedded translational studies within clinical trials to improve understand.

### Is AF a causal actor or epiphenomenon of the adverse outcomes associated with critical illness?

Causal links between adverse outcomes and the onset, duration, and intensity of AF during acute and critical illness, as well as a clear understanding of the natural history of this arrhythmia, require further clarification. While worse outcomes such as mortality are clearly associated with AF during critical illness, it remains unclear if AF is part of the causal pathway to adverse outcomes, or if AF is merely an epiphenomenon reflective of illness severity. For example, an effective randomized study of AF prevention which demonstrates a difference in clinical outcomes might signify that AF itself acts as a treatable target, or that AF denotes a deranged physiological state responsive to the studied intervention (e.g., beta-blockers may attenuate sympathetic overdrive). Thus, while randomized trials directed at preventing/treating AF may provide clinically useful interventions, interpretations of AF trials must be careful to evaluate pathways in which treatments directed at AF may influence the underlying disease state and alter the course of illness separate from treatment of AF alone.

**Fig. 1:**
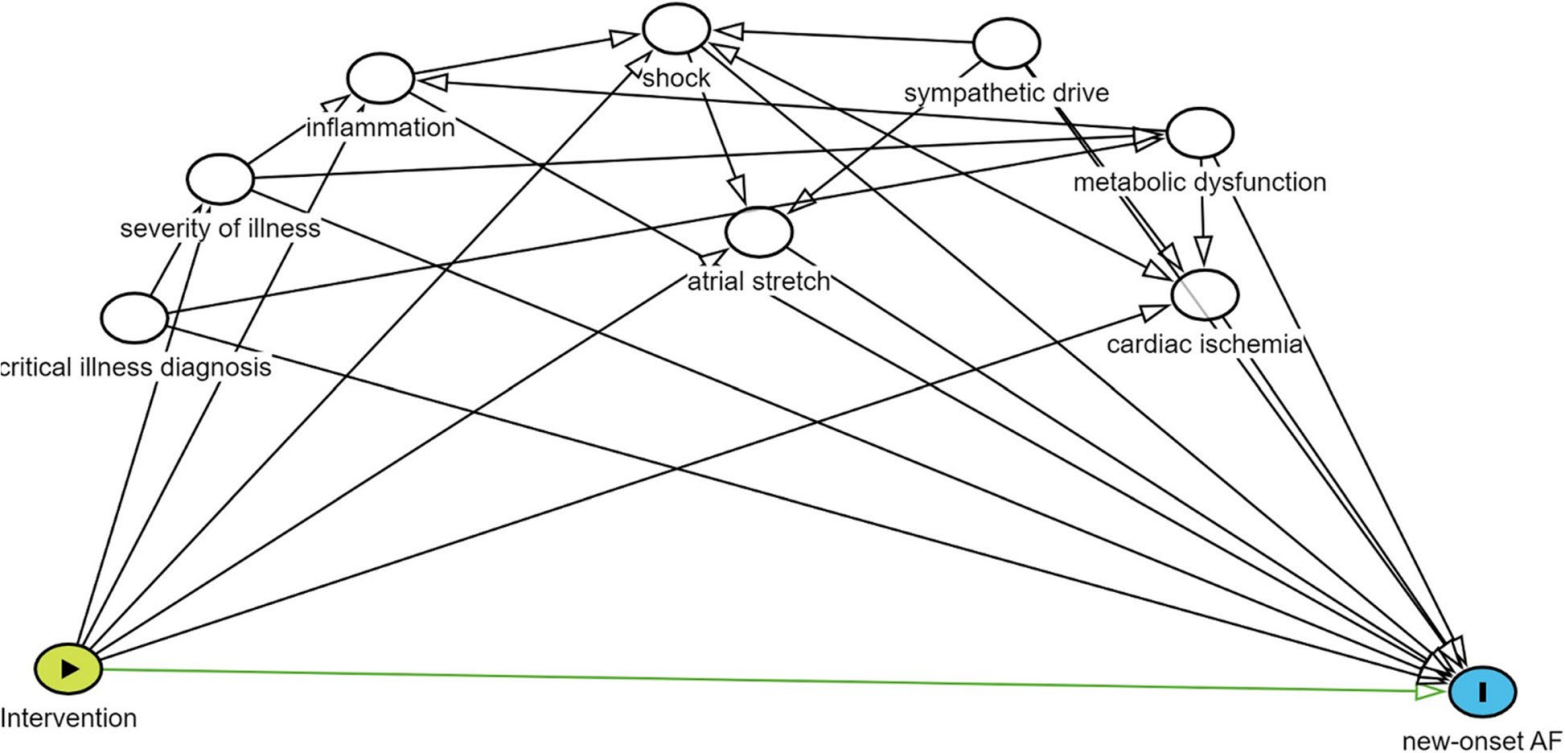
Example of a direct acyclic graph evaluating an prophylactic intervention for new-onset atrial fibrillation

- *Research impact* - It is difficult to determine the attributable risk of AF (chronic or new onset) to short and long-term outcomes without careful consideration of mediators and effect modifiers.
- *Recommendations* - Future research should explore causal relationships during the design phase of the study using causal diagrams or directed acyclic graphs (DAG). These results will inform the appropriate study design and will improve understanding of causality between AF and outcomes (Fig. 1).

### What are the important outcomes linked to AF in acute and critical illness?

**Fig. 2:**
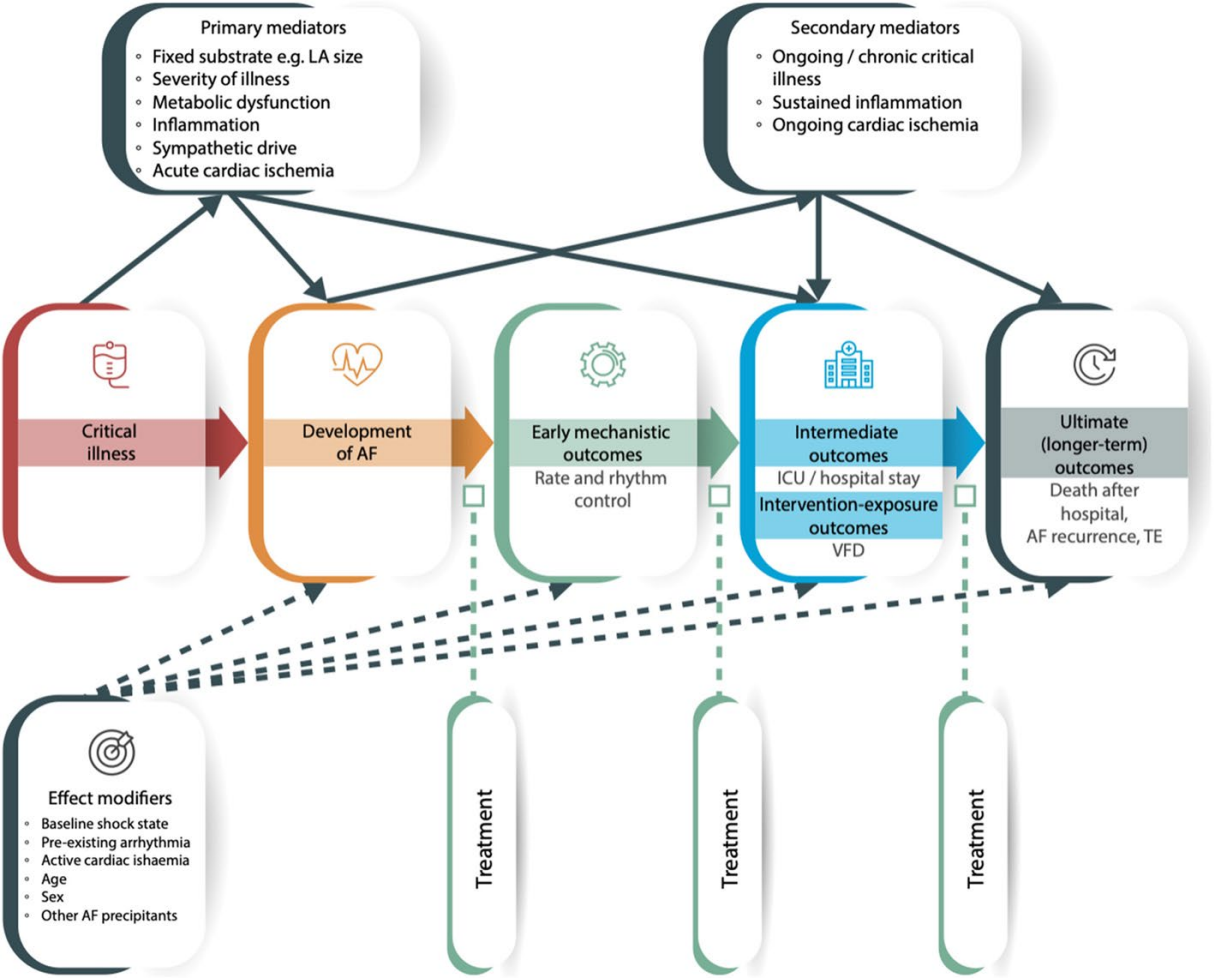
Exposure / outcome diagram for the development and outcomes of AF in critical illness identifying the relationship between critical illness and the development of AF and early, intermediate, and long-term outcomes, primary and secondary mediators (solid lines), possible effect modifiers (dashed lines), and differing potential treatments regarding the disease stages and outcomes. Square arrowheads represent potential treatment effect. AF = atrial fibrillation, ICU = intensive care unit, VFD = ventilator-free days, TE = thromboembolism

Currently there is substantial variation in outcomes measured during interventional trials of AF management, with the most common being rate and/or rhythm control, successful cardioversion, maintenance of normal sinus rhythm, thromboembolic events, heart failure, length of stay, quality of life, and mortality [1, 29]. Follow-up time periods are an important consideration; for example, an episode of AF during critical illness may contribute greater risk to 28-day mortality than 5-year mortality, and the impact of a treatment may have both immediate and long-term consequences. Furthermore, the desired goal of treatment (i.e., rhythm versus rate control) should also be considered as they may differ between patients with and without AF associated hemodynamic instability, for example. Patient-important outcomes are a consideration, as they are more likely to be concerned with risk of stroke, quality of life, and death rather than outcomes such as heart rate or rhythm control (Fig. 2).

Additionally, outcomes related to resource utilization must be considered, as the development of AF has been associated with increased length of hospital stay and costs [30]. There is potential for important system level outcomes linked to prevention and treatment strategies.

- *Research impact* - Current AF research in the acute and critical illness setting often uses outcomes that are not clearly related in a casual manner, lack demonstrated clinical importance, or are not patient centred. The outcomes being measured are not consistent across trials making comparisons difficult.
- *Recommendations* - There is a need to develop and weight a core outcome set to define mechanistically important and patient-important outcomes of AF in acute and critical illness [31]. Studies should be designed with this outcome set in mind, and patient input must be integrated into trial development, including patient reported outcome measures.

### What factors should be considered to enrol patients into time-sensitive AF trials in challenging environments?

Acutely ill patients with AF are a challenging group to recruit into trials because their clinical condition is perceived to require prompt intervention, and treatment often occurs before there is opportunity to enroll them in a clinical study. Clinicians may be resistant to enroll hemodynamically unstable patients into studies where they perceive potential for a treatment to worsen the patient’s clinical condition. Systematic detection of the arrhythmia in areas where patients are unlikely to have continuous monitoring presents a unique challenge, limiting ability to recruit ward patients into time-sensitive trials. Recruitment of patients in the emergency department is challenging due to pace of care and movement of patients to different care areas and clinical teams. Additionally, each environment presents unique challenges regarding the patient’s ability to consent, availability of next of kin, monitoring and treatment options, familiarity with research processes, as well as timely access to research coordinators.

- *Research impact* - There are many missed opportunities to include eligible AF patients in research studies. These factors may lead to biased recruitment into trials.
- *Recommendations* - Studies of AF in acute and critically ill patients would benefit from systems of clinical care where research is embedded into clinical practice [32]. This includes embracing a culture where nurses, physicians, and trainees recognize and feel empowered to identify patients who would be potential candidates for studies, and lobbying for buy-in from stakeholders who oversee hospital policy to ensure such research models can exist. Consent models may need to be adapted, particularly for low-risk interventions, to a deferred consent model where possible and ethically appropriate to ensure that patients are not denied the opportunity to participate in clinical research. Involvement of patient and public partners at the study design phase can provide valuable insight into the acceptability of deferred consent models [33].

### What is the potential impact of enhanced interprofessional clinical and academic collaboration in AF research?

Current guidelines highlight the need for an interdisciplinary approach to the management of AF [34, 35]. AF in acute and critical illness is not confined to a physical location and is recognized and treated at several phases during a patient’s clinical journey [36]. Lack of collaboration of providers along this patient pathway may lead to variable and sometimes conflicting treatments, poor communication around brief episodes of arrhythmia and missed opportunities for long-term follow-up and risk mitigation [37]. From a research perspective, an interdisciplinary approach offers opportunity for earlier engagement in the clinical course, opportunities for obtaining biologic and physiologic samples in the pre- and post-AF phases of illness, continuation of interventions initiated early in the clinical trajectory, and long-term follow-up and measurements of outcomes at various time points [38].

Community hospitals are more likely to be located in suburban and rural communities and are more likely to serve older patients, racialized communities, and patients with lower socioeconomic status and reduced access to subspecialized care [39]. Engagement of colleagues outside of academic settings allows for participation of a more diverse group of patients, representing populations who may be underrepresented in clinical trials, and would improve the generalizability of study results.

- *Research Impact* – AF is a syndrome that occurs at various time points in a clinical course and in different locations within and outside the hospital setting. Interdisciplinary collaboration is required to optimize enrollment, data collection, interventions, follow-up, and generalizability.
- *Recommendations* – Interdisciplinary collaboration should occur early during development of the study design, with consideration of important potential time points and opportunities for long term follow-up. Special consideration for the unique challenges in various clinical settings should be anticipated, and funding requests should reflect the need for optimal infrastructure, including research coordinator support across various clinical areas, to coordinate recruitment and data collection. There is a need for an integrated knowledge translation plans where findings of studies conducted in acutely ill populations are dispersed widely and not limited to intensive care audiences. Community hospital sites should be involved early in study planning to ensure trials are feasible and are associated with adequate funding to support research in non-academic settings.

### What technologies/innovations could improve AF research?

There are several promising technologies and research innovations that have the potential to advance the study of AF in acute and critical illness. Perhaps most important is the opportunity to embed clinical trials into clinical care platforms, facilitated by EMRs and databases, improving efficiency and generalizability of clinical trials. Use of artificial intelligence (AI) is increasing in medicine and actionable AI could be envisioned in prediction of the arrhythmia, non-invasive detection of the arrhythmia, and prediction of long-term outcomes [40–42]. Bioinformatics could improve our understanding of the potential mechanisms and outcomes of AF in critically ill patients [43]. Echocardiography may provide valuable insight into the etiology of AF in critically ill patients, heterogeneity of treatment effects, and improve prediction of long-term outcomes such as AF recurrence. Echocardiography may play a role in patient recruitment to studies allowing enrichment of patient populations and increased treatment precision [44–50]. Echocardiographic images can provide high-fidelity physiologic data to improve AI prediction and outcome models [51]. Novel technologies, including remote wireless patch-based monitoring systems [52] and smart watches [53] can provide continuous high granularity data such as electrocardiographic waveforms and vital signs to inform about predictors and risk factors, as well as treatment response, with potential to improve long-term follow up, especially for patients who are at community centres or unable to travel to research sites.

- *Research Impact* – New technologies and innovations could be leveraged to improve recruitment and generalizability in clinical trials, to enrich study populations, to deliver care with increased clinical precision, and to improve long-term follow up. However, these technologies require research software within clinical care platforms, collection of high quality serum and tissue samples and imaging and physiologic waveform data, significant computing power, development and maintenance of granular databases, and data-sharing capabilities, rendering it costly and labourious to implement into new studies and existing platforms.
- *Recommendation* – Lobbying of stakeholders is necessary to embed AF research into clinical care and to develop and maintain reliable and accessable databases. Collection of physiologic data and biologic samples should be embedded in clinical trials to increase efficiency in the collection of samples for AI and bioinformatics research. Funding agencies need to be aware of the explicit benefits of novel technologies to fund their use in clinical trials for improved monitoring and follow-up.

## Future directions

There is a need to continue the discussions initiated at the Symposium on Atrial Fibrillation in Acute and Critical Care and address issues that were identified. Future meetings would aim to include more AF researchers and a broader interdisciplinary group, specifically increased participation with colleagues from nursing and patient and family advisors. The concentration of these meetings would include the development of definitions of AF (possibly by a Delphi process), the development of a core outcome set, and targeted advocacy from the group to improve research processes, funding, and data-sharing agreements.

## Conclusions

Study of AF in critical illness has unique challenges due to the nature of the arrhythmia, the difficulties conducting time-sensitive studies in critical illness, and the disconnection of clinical specialities in the evaluation and treatment of AF during a patient’s clinical journey. Efforts to resolve these issues are necessary to improve patient outcomes and should focus on clear and prognostically important definitions, patient-centred core outcomes, integration of translational studies into clinical trials, and advocacy for new and innovative technologies. Most importantly, an cross-disciplinary collaboration must be pursued to allow the optimization of research efforts and ensure patients who develop AF during their acute and critical illness are able to participate in clinical studies to inform evidence-based care.

## Data Availability

Not applicable.

## Abbreviations

AF: Atrial fibrillation
AI: Artificial intelligence
DAG: Directed acyclic graphs
NOAF: New-onset atrial fibrillation
ICU: Intensive care unit
